# Enhancing heart valve disease surveillance: a quality improvement project demonstrating cost-effective triaging and Clinical Scientist-led services to improve patient care

**DOI:** 10.1186/s44156-025-00096-x

**Published:** 2025-10-10

**Authors:** Emily King, Richard Clements, Nathan Proudlove

**Affiliations:** 1 Musgrove Park Hospital, Somerset NHS Foundation Trust, Taunton, UK; 2 Alliance Manchester Business School, University of Manchester, Manchester, UK

**Keywords:** Cardiac science, Heart valve disease, Heart valve clinic, Transthoracic echocardiography, Clinical scientist led clinics, Quality improvement, Process mapping, Statistical process control, Productivity

## Abstract

Heart valve disease (HVD) is increasing in prevalence in the UK due to the ageing population, placing greater demands on diagnostic heart valve clinics. While many services recognise the need to improve efficiency and standardisation, initiating and implementing excellent quality improvement projects (QIPs) remains a challenge. Particularly for time-constrained service leads with limited resources and experience. This QIP describes a practical, replicable intervention to enhance HVD surveillance services using structured process mapping, root cause analysis and iterative Plan-Do-Study-Act (PDSA) cycles. Key issues identified included premature surveillance bookings, delays in result dissemination, and underutilisation of Clinical Scientists, contributing to inefficient workflows for consultant cardiologists and unnecessary visits for patients. The project introduced guidelined-aligned surveillance intervals, a refined triaging system, and a parallel Clinical Scientist-Led Valve Clinic (SLVC) pathway. Over four PDSA cycles, adherence to British Society of Echocardiography surveillance guidelines improved from 33% to 88%. Mean surveillance earliness was reduced from 3.4 months to 1.2 months in the Cardiologist-Led Care (CLC) pathway and to 0.5 months in the SLVC. Result dispatch times also improved significantly, with SLVC letters averaging 1.4 days (93% dispatched within five working days). A simple cost model suggested a 21% cost reduction if the SLVC pathway was scaled across the surveillance population, with estimated productivity gains of 12% in CLC and 17% through the SLVC, yielding a total projected improvement of 15%. These gains are attributed to optimised triaging, reduced overprocessing and the lower per-patient cost of SLVC delivery. This paper provides a detailed, real-world example of an adaptable QIP. It offers a practical framework for improving HVD surveillance services in resource-constrained settings while achieving measurable clinical and operational benefits.

## Introduction

The prevalence of heart valve disease (HVD) is rising in the UK due to an ageing population. Simultaneously, consultant cardiologists face increasing clinical demands in addition to the national shortage of professionals capable of performing transthoracic echocardiography (TTE) [1, 2]. This workforce shortage of Clinical Scientists and cardiac physiologists stems from insufficient training capacity, high workload, limited career progression and low job satisfaction [3]. 

At Somerset NHS Foundation Trust (SFT), HVD surveillance services are based at Musgrove Park Hospital (MPH), where outpatient follow-up has traditionally followed a cardiologist-led care (CLC) model. An audit of surveillance TTE requests between 19th October and 27th November 2023 revealed multiple inefficiencies, including major deviations from British Society of Echocardiography (BSE) guidance, with some patients being recalled months earlier than necessary. Additional issues included duplicate or absent TTE bookings and administrative delays, such as slow dispatch of results to patients and their general practitioners (GPs).

In response, two principle change ideas were developed: the implementation of department-specific triage protocols aligning with BSE surveillance intervals, and the establishment of a Clinical Scientist-Led Valve Clinic (SLVC) to manage an appropriate sub-group of patients. This model aimed to reduce the burden on consultant-led services and improve consistency in clinical pathways.

While there is a growing body of literature on the structure and benefits of valve clinics [4–7] including the recent GIRFT report endorsing extended roles: “cardiac physiologists should be undertaking extended roles such as valve surveillance clinics”(p.22) [8], evidence demonstrating the impact of quality improvement projects (QIPs) in this context remains limited [9]. Some QIPs have focused on reducing unused TTE appointments [10, 11] or expanding Clinical-Scientist-led transoesophageal echocardiogram services [12]. However, few have addressed structural improvements to HVD surveillance.

The aim of this QIP was to improve the consistency and efficiency of care for patients with mild to less-than-severe HVD. This involved of two main SMART (Specific, Measurable, Achievable, Realistic, Timely) goals to be completed by June 2024:

1. increase the percentage of patients booked no earlier than one month before the BSE-recommended interval to 90%, with a mean earliness of less than one month.
2. Reduce average delay in dispatching result letters to fewer than five working days.

And two subsidiary goals:

3. Reduce duplicate echocardiogram bookings to less than 1%.
4. Achieve a cost reduction of more than 20% in HVD surveillance.

**Fig. 1:**
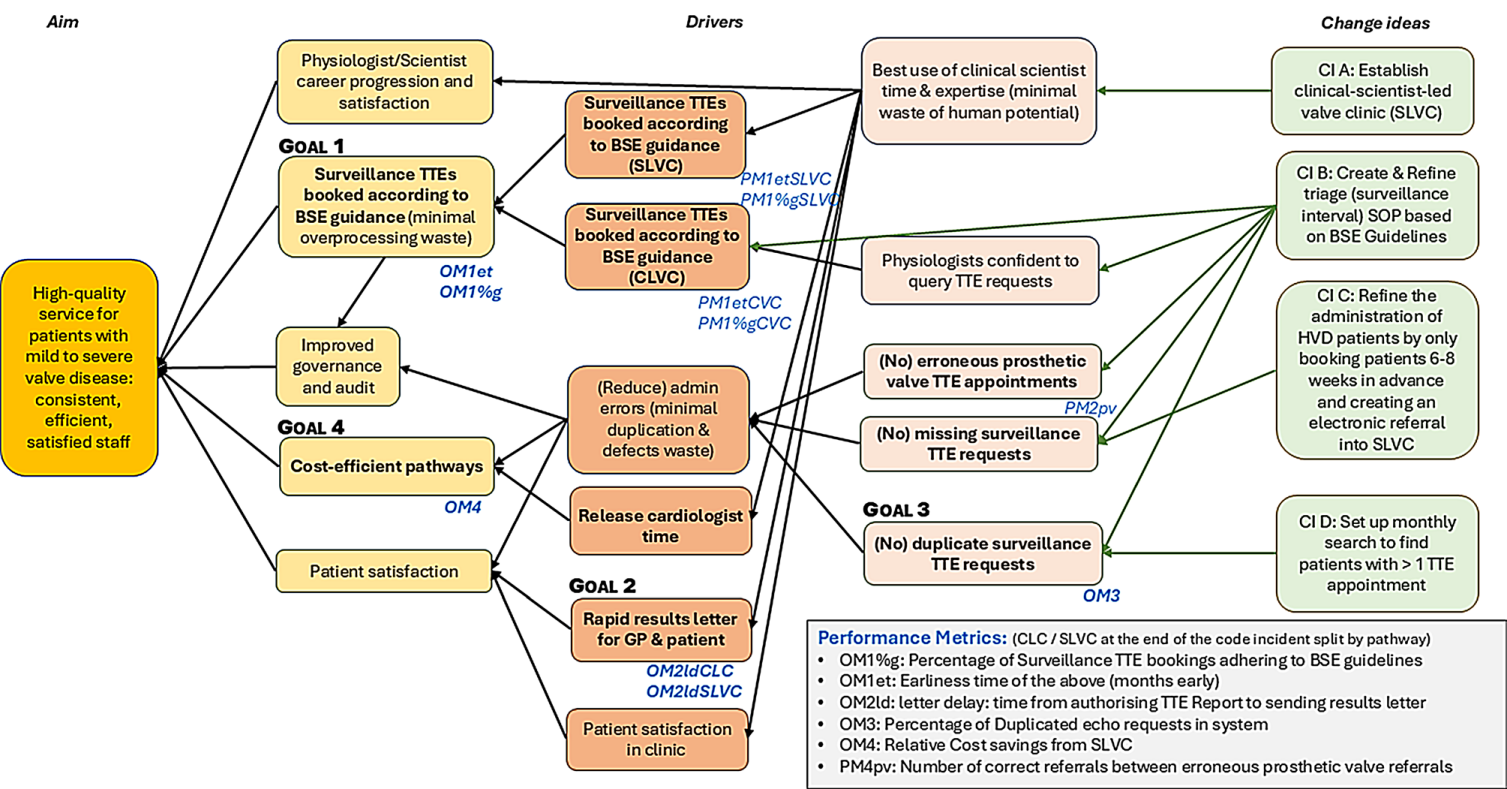
Driver diagram for quality improvement project

The project logic is shown in the driver diagram [13, 14] (Fig. 1). The QIP was structured using the Model for Improvement (MfI) [13, 15] which centres on three key questions: ‘What are we trying to accomplish?’, ‘How will we know a change is an improvement?’ and ‘What changes can we make that will result in improvement?’ This framework guided the development of change ideas (CIs) and their iterative testing through Plan-Do-Study-Act (PDSA) cycles. The MfI has been used successfully to improve performance in NHS clinical sciences departments, specifically in physical sciences [16], life sciences [17–21], and physiological sciences [22, 23], including cardiac science [10, 12]. This report is structured in line with the revised Standards for Quality Improvement Reporting Excellence (SQUIRE 2.0) guidelines [24]. 

## Background

SFT operates two district general hospitals, with MPH serving as the larger site and providing specialist cardiology services for a population of approximately 544 000 people. The demographic of this region is relatively older than the national average, with a median age of 47 years compared with 40 for England and Wales [25]. 

In the UK, 11% of the population over 65 years old are affected by HVD [26], and this proportion is expected to increase further [27]. Within SFT’s catchment, the number of individuals aged over 65 with cardiovascular disease is projected to rise from 50,012 to approximately 67,250 by 2040 [28]. This growing burden necessitates proactive and efficient HVD surveillance systems.

TTE remains the cornerstone of non-invasive assessment for HVD, and surveillance valve clinics are endorsed as a key component of specialised care [29]. Traditionally at SFT, these clinics have followed a CLC model: patients attend an appointment for a TTE performed by a Clinical Scientist or cardiac physiologist, and results are subsequently reviewed by a cardiologist who communicates findings and next steps.

An alternative model, SLVC, offers numerous advantages. These include: a standardised and consistent approach to care for patients with less-than-severe HVD; fewer hospital visits; lower per-patient cost; opportunities to expand Clinical Scientists’ roles; and enhancing motivation and retention within the workforce [8, 30]. Appropriate patients for SLVC include those with: less-than-severe left-sided HVD in patients with normal left ventricular function, valve repairs, tissue aortic valve replacements and transcatheter aortic valve implantations.

In such cases, Clinical Scientists can conduct history-taking, perform the TTE, assess symptoms and valve progression, formulate a management plan (including discharge, continued surveillance, or referral to a cardiologist), and communicate results directly to the patient. This ‘one-stop shop’ model improves efficiency and patient experience, as demonstrated in other Clinical-Scientist-led services [31, 32]. Regulation by the Health and Care Professions Council (HCPC) was deemed important for those undertaking advanced practice at SFT, therefore SLVC by definition will be run by professionals who are registered as Clinical Scientists with the HCPC.

By 2014, approximately 21% of UK hospitals had established HVD clinics, with 24% of these led by ‘sonographers’ [33]. A 2021 British Heart Valve Society survey reported that 68% of UK hospitals have a valve clinic, although significant variation in practice remains [9]. SLVCs represent a scalable and clinically sound model to deliver expert-led, patient-centred care, ensuring timely diagnosis and intervention, and relieve pressure on cardiologist-led services.

## Method

### Baseline Analysis

**Fig. 2:**
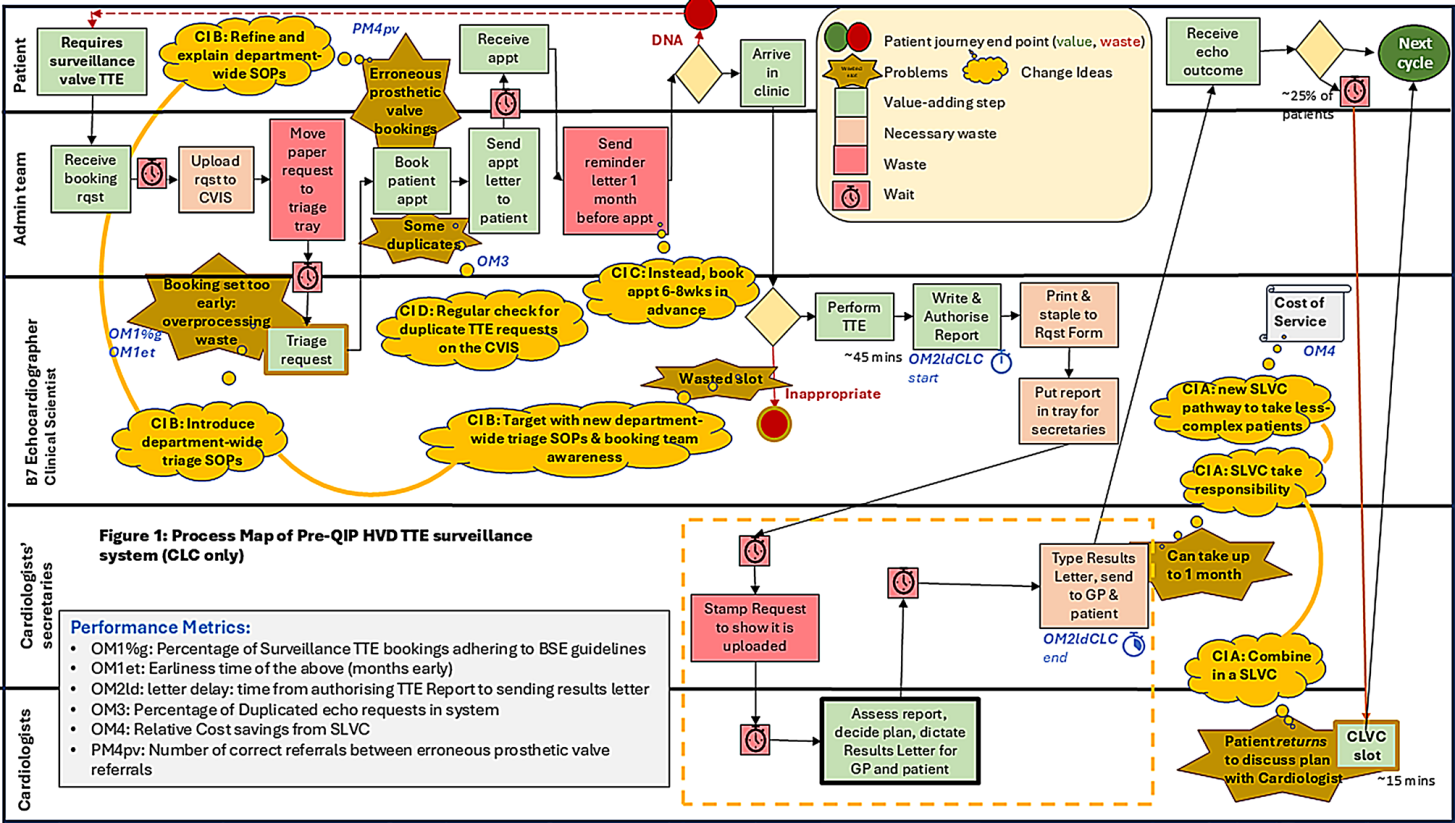
Process map of pre-QIP HVD TTE surveillance system (CLC only)

Initial baseline analysis focused on patients undergoing surveillance TTEs under the traditional CLC model between 19th October and 27th November 2023. A process map [13, 34, 35] of this care pathway was created to identify inefficiencies and sources of waste (Fig. 2). In this model, patients received a surveillance TTE from a Clinical Scientist or cardiac physiologist, with the report subsequently reviewed by a cardiologist. This resulted in a letter being written to the patient and their GP, sometimes followed by a clinic appointment. Several delays and inefficiencies were identified in this sequence ((Fig. 2 highlights wastes (red shading) and problem issues (brown stars)).

**Fig. 3:**
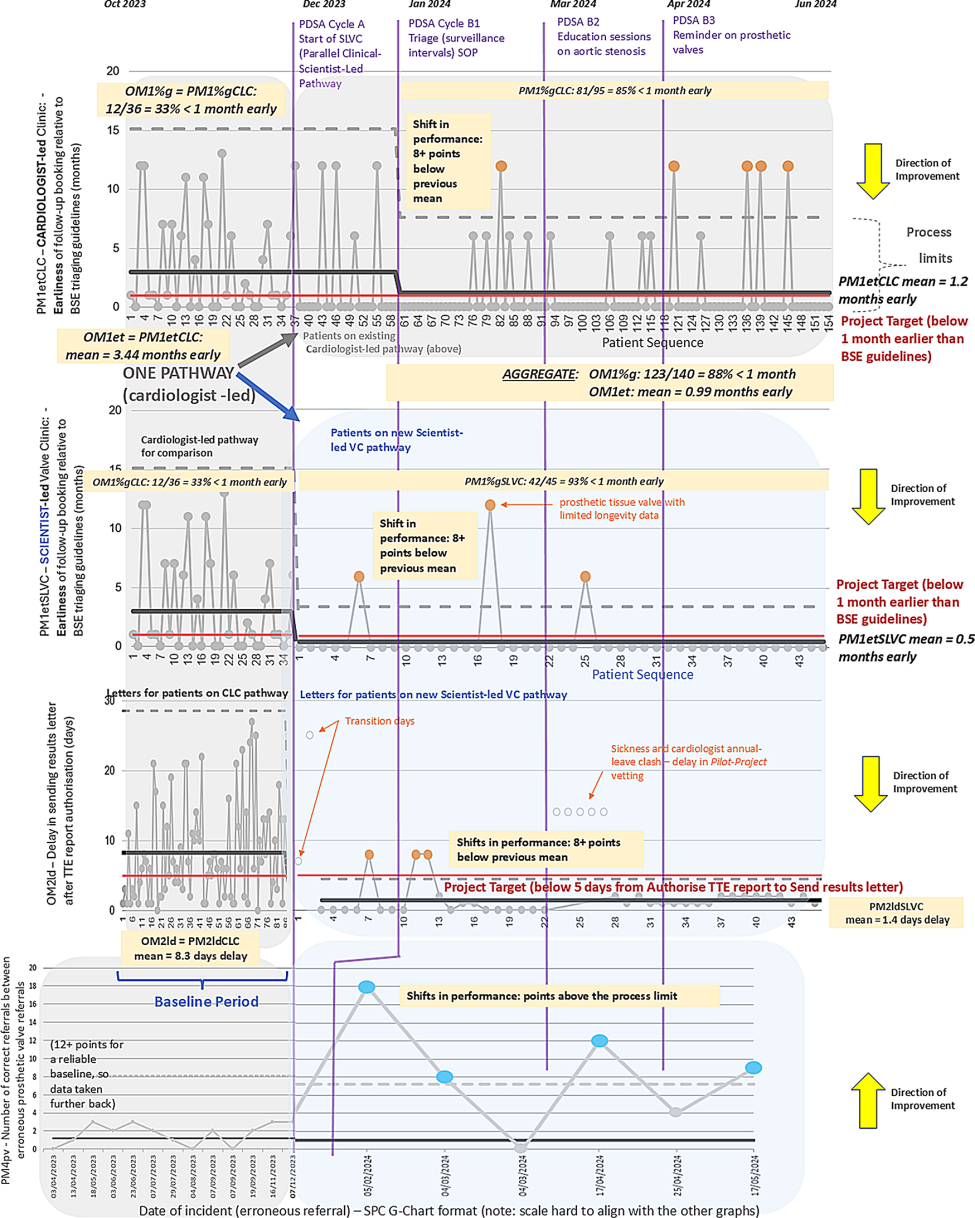
Metrics over time: SPC charts

A key issue was inconsistent and premature triaging of patients for surveillance TTEs, often scheduled significantly earlier than recommended by BSE guidance. This early scheduling constituted overprocessing [15, 36], unnecessarily consuming clinical capacity and patient time. Surveillance earliness was quantified as a core outcome metric (OM): OM1et (mean earliness in months before the guideline-recommended interval) and OM1%g (percentage of patients booked no more than one month early). Baseline data demonstrated a mean earliness of 3.44 months with significant variability (Fig. 3). These timeseries data are shown in Fig. 3 as statistical process control (SPC) format graphs [13, 15]. 

From the first PDSA cycle, SLVC and CLC metrics were reported separately and treated as parallel process metrics (PMs). The goal was to reduce mean earliness to less than one month. Root cause analysis revealed that the triaging echocardiographers were not applying a standard triaging approach and were inconsistently referencing BSE guidelines. Cardiologists were also sometimes unaware of the guideline detail.

Another issue was delay in dispatching result letters to patients and GPs following TTEs. The baseline mean delay was 8.25 days, with a range of 0–28 days (Fig. 3). This was captured as OM2ld. This metric, too, was later split between CLC and SLVC comparison.

Booking system inefficiencies were also identified, including duplicate and missing appointments. The local IT platform had limited search functionality, requiring time-intensive review to identify duplication. Although the system could not track when duplicates were created, it was possible to estimate the rate of duplication (OM3) before and after intervention.

**Fig. 4:**
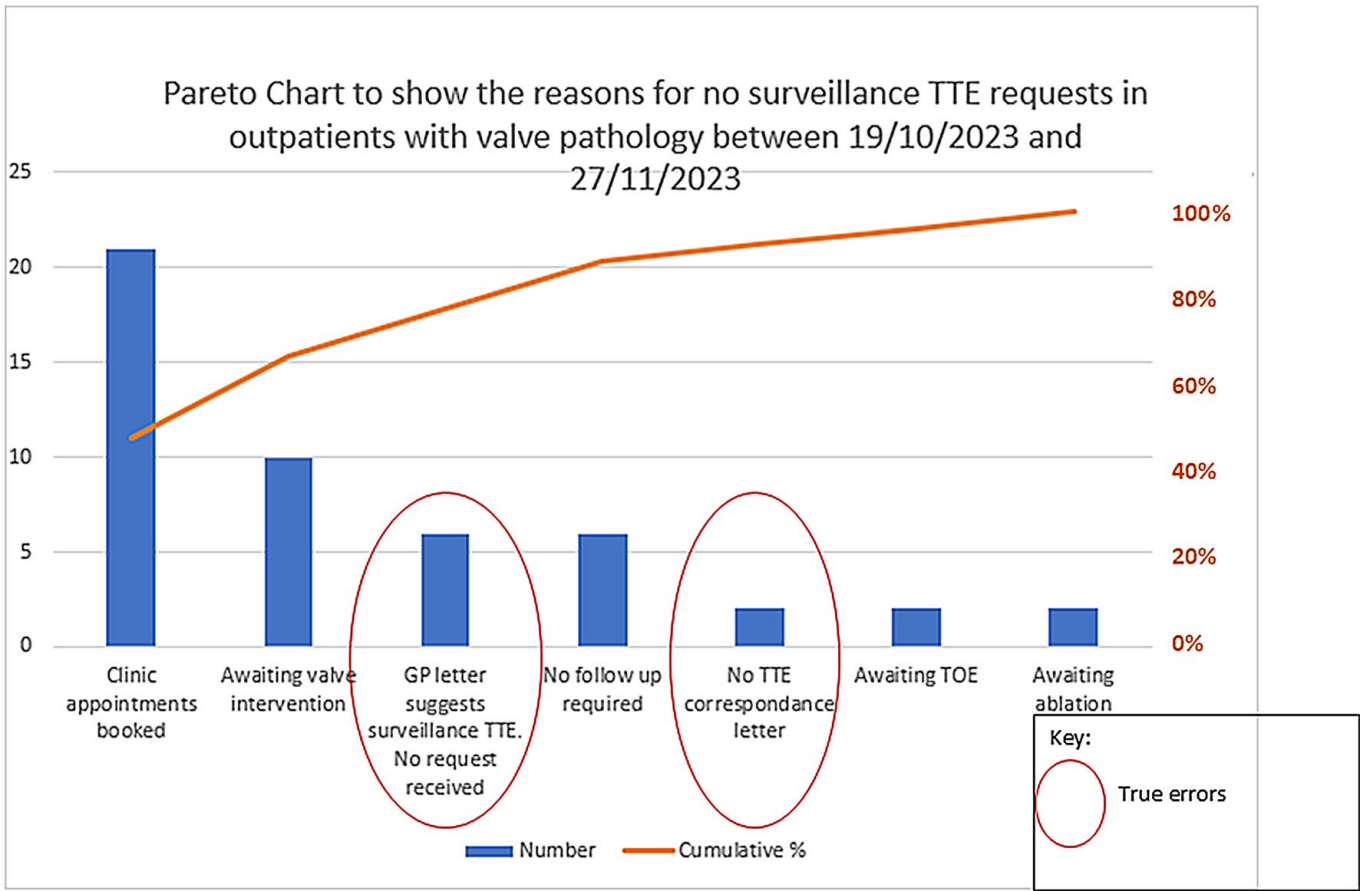
Pareto chart: missing bookings

Analysis of cases with missing surveillance TTE bookings found that 16% represented true omissions. These were often attributable to cardiologists not requesting follow-up scans, incomplete paperwork, or failure to act on prior TTE results (Fig. 4). It was anticipated that SLVCs would mitigate such errors by streamlining triage and reducing handovers.

**Fig. 5:**
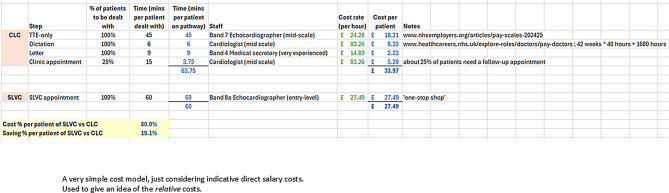
Simple cost comparison

Relative cost savings (OM4) were estimated by comparing the per-patient cost of CLC and SLVC pathways (Fig. 5). Based on 2024/25 NHS tariff rates [37], CLC follow-up cost is £106 and a TTE for an adult is £93, equating to £199 per patient. The SLVC model combined the TTE with 15 min of additional Clinical Scientist time. A significant challenge with direct cost comparison exists because there is no direct tariff for SLVCs. In light of the GIRFT report [8] advocating for these services, this is an issue that requires addressing. Regardless, an internal costing based on staff time suggested that SLVCs could deliver care at approximately 81% of the cost of CLC, even without factoring in productivity benefit (Fig. 5).

Finally, surveillance of patients with prosthetic valves, despite known valve durability, was frequently scheduled too early. PM4pv tracked these cases on a SPC G chart [38] (Fig. 3, bottom), quantifying the number of correct triages between errors.

### Design of the interventions

Following review of the process map, driver diagram, and root cause analysis, a multidisciplinary team (MDT) consisting of cardiologists, echo service leads and the outpatient booking manager, developed four primary CIs to address the identified inefficiencies (Fig. 2):

A. Establish a SLVC.
B. Create and use a set of triaging Standard Operating Procedures (SOPs) for the whole department for booking surveillance TTEs at appropriate intervals.
C. Refine the administration of the booking and referral system for HVD patients, booking patients 6–8 weeks in advance and creating an electronic referral into SLVC.
D. Create an automatic search on the system to find patients with more than one booked appointment.

#### CI A: establish a SLVC

Building on models already adopted in other NHS trusts, the SLVC aimed to reduce consultant workload and enable Clinical Scientists to manage lower-complexity cases directly. The SLVC was designed to offer eight patient slots per day (compared with ten under CLC), offset by reduced TTE volumes through appropriate triaging. SLVC inclusion criteria were defined collaboratively and included patients with:

- less-than-severe left-sided aortic or mitral valve disease.
  - *and* no more than mild right-sided valve disease.
  - *and* normal left ventricular systolic function.
- left-sided tissue valve replacements *or* left-sided valve repairs.
  - *and* normal left ventricular systolic function.

During the SLVC appointment, the Clinical Scientist would perform the TTE, take a brief clinical history, perform a targeted examination, evaluate valve progression using TTE, discuss the management plan with the patient, and write the results letter for the GP and patient. Auscultation was not routinely included as all patients received a full TTE. This “one-stop” model reduced unnecessary visits and accelerated communication of results. There is evidence that this is beneficial, allaying patients anxiety waiting for results and allowing them to make informed decisions [39]. 

During the pilot, all letters were vetted by a cardiologist as part of a quality assurance process. Exclusion criteria were established for more complex patients to remain under the CLC pathway. This was created in conjunction with the cardiologists to prevent capacity issues. Such as, more patients being referred into the service than can be maintained and avoiding taking up their time making case-by-case decisions on grey-area cases. The criteria will likely be adapted as the Clinical Scientists get more experienced leading the clinic. Competency requirements for SLVC leaders included:

- Minimum of three years BSE Level 2 TTE accreditation.
- Completion of a recognised clinical history-taking course.
- Observation of at least ten clinical histories by a consultant cardiologist.
- Signed-off extended practice competency protocol.

**Fig. 6:**
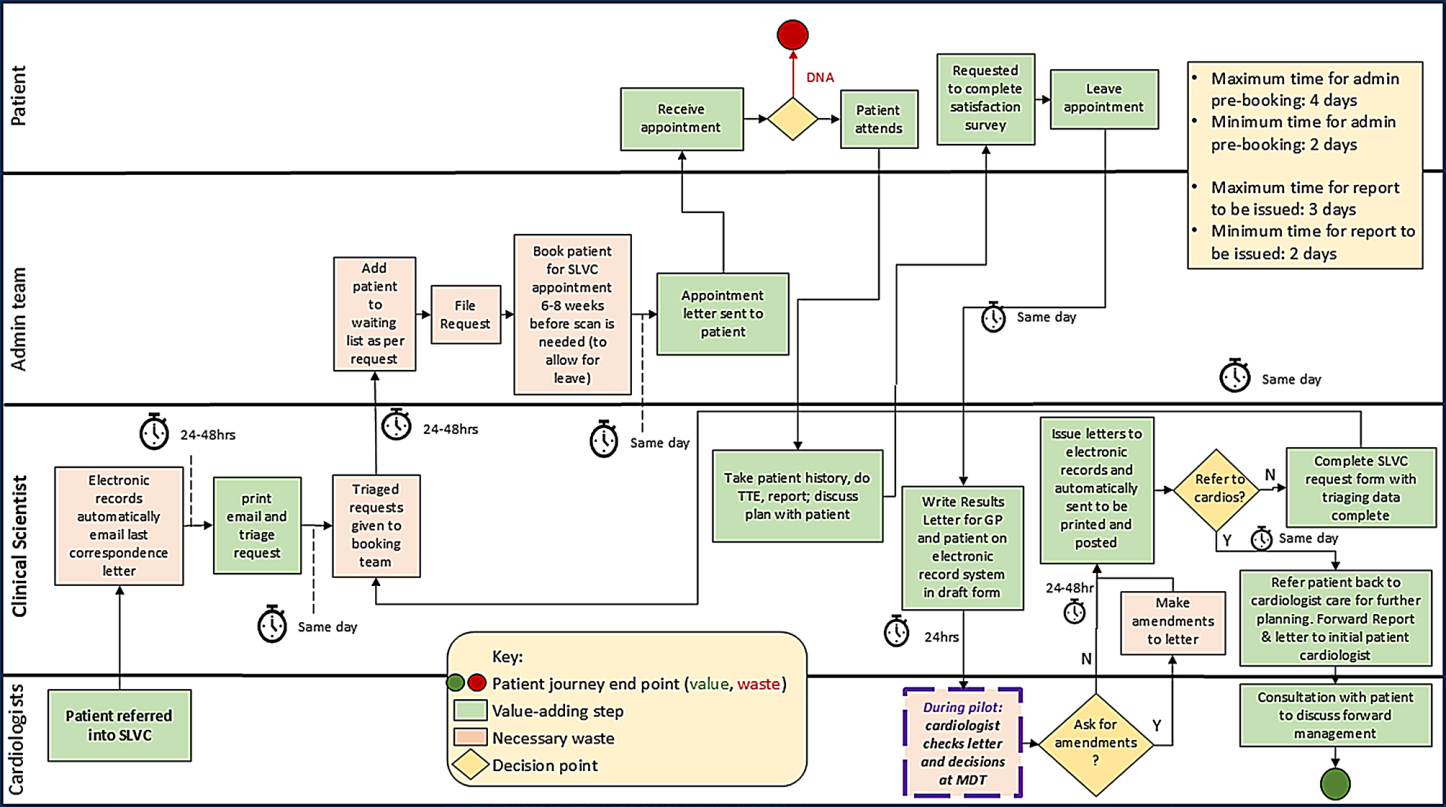
Process map of the new SLVC pathway

Figure 6 illustrates the process flow for this pathway. Contrasting this with the number of job handoffs between staff groups in the CLC (Fig. 6) suggests greatly reduced opportunities for omissions and delays.

#### CI B: implement department-wide SOPs for surveillance triaging

Triage consistency varied significantly across the department. The new SOPs were based on BSE guidelines and clarified appropriate surveillance intervals. They aimed to standardise practice for both SLVC and CLC patients.

A particular focus was placed on moderate aortic stenosis (AS) cases. At the time of the QIP, AS surveillance was dependent on peak aortic velocity (Vmax). The SOP distinguished between patients with Vmax between 3.0 and 3.4 m/s (recommended follow-up 18–24 months) and those with 3.5–3.9 m/s (12–18 months). Many cardiologists had defaulted to 12-month surveillance for all moderate cases, which resulted in premature bookings. Educational sessions reinforced SOP use and clarified prosthetic valve triage procedures.

#### CI C: refine administration and introduce electronic referrals to SLVC

Administrative inefficiencies were linked to long-term advanced booking and paper-based referrals. To address this, surveillance TTEs were scheduled only 6–8 weeks in advance. An electronic referral mechanism was created within the local electronic medical record system. When a consultant referred a patient to SLVC, a copy of the patient’s clinic letter was emailed to the SLVC team. This acted as a referral and was sorted into folders based on the intended appointment month. The admin team then scheduled appointments from this pool on a rolling monthly basis.

An early challenge emerged when some ineligible patients were mistakenly referred. As the referral letter was simultaneously sent to the patient and the SLVC team, unsuitable referrals could not be intercepted beforehand. The workaround involved rejecting the referral and informing the consultant, who would then follow up directly with the patient.

#### CI D: develop a monthly search system for duplicate bookings

A basic query was created within the Cardio-Vascular Information System to identify patients with more than one booked appointment. The data was exported to Excel, and duplicates were flagged using conditional formatting. Although no baseline data was available, anecdotal reports from staff confirmed the prevalence of booking duplication. The search is now run monthly to prevent unnecessary appointments and resource use.

**Fig. 7:**
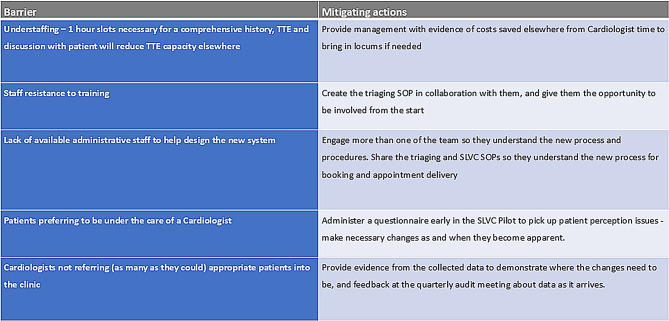
Potential barriers and mitigating actions for the proposed changes

It is very common to encounter potential difficulties and barriers to CIs [40], these barriers and corresponding mitigation strategies were identified, and are summarised in Fig. 7.

### Testing the interventions

The two primary interventions (CIs A and B) were tested using iterative PDSA cycles to evaluate the SLVC model and refine departmental triaging practices. The two administrative interventions (CIs C and D) were implemented as “just do it” (JDI) [13] changes due to their straightforward nature. The PDSAs and their results are summarised in Table 1; Fig. 3.

**Table 1:** Summary of Plan-Do-Study-Act (PDSA) cycles

Cycle	Plan/Prediction	Do	Study	Act	Time
Baseline	There are differing methods of triaging echocardiography requests	Assess the number of valve patients triaged according to BSE guidelines. Assess for the number of duplicate requests in the system for future appointment	Previous system [*CLC only*]: OM1%g = PM1%gCLC: 12/36 **= 33% < 1 month** early OM1et = PM1etCLC: mean = **3.44 months early** OM2ld = PM2ldCLC mean = **8.3 days delay to letters**	System with much waste: design and experiment with feasible change ideas - to pilot and evaluate an SLVC and introduce department-wide SOPs for triage of surveillance TTE booking intervals	7 weeks
PDSA Cycle B1	CI B: An approved triaging SOP will give those triaging the confidence to challenge requests from cardiologists if they fall outside the guidelines	Introduce SOP based on BSE guidelines and local protocols for interval of surveillance TTE	*CLC*: (for whole of rest of pilot period) PM1%gCLC ***81/95 = 85% < 1 month early*** *PM1etCLC* ***mean = 1.2 months early*** *SLVC*: sustaining performance	*CLC*: materially better & sustained performance, though not meeting the targets. Behaviour less haphazard, but some patients being booked for 6 or 12 months early. Apparent that there is an assumption amongst some staff that all patients with moderate aortic stenosis need to be scanned every 12 months	Jan 24 4 weeks
PDSA Cycle B2	CI B: Reduce confusion about assessment of moderate aortic stenosis will improve consistency	Educational sessions for dept on BSE guidelines for moderate aortic stenosis: explain why the patients are triaged according to peak velocity, rather than severity definition		Apparent that there is some confusion amongst some staff about criteria for triage of prosthetic heart valve cases	Feb 24 7 weeks
PDSA Cycle B3	CI B: Variations will occur particularly with prosthetic heart valve patients due to the lack of clarity regarding prosthetic valves with known durability	Document the recommended follow up period for each prosthetic valve type and distribute the information to others running SLVC.	Material reduction in rate of erroneous in prosthetic heart triaging (SPC G chart Fig. 3, bottom) OM3 = **0.3% duplicate bookings** OM4 = **19.4%** relative cost saving	Triaging SOPs effective in reducing these, but more needs to be done. At the end of the pilot, assess consultant time saved and percentage cost saving between two systems.	Mar 24 13 weeks

#### PDSA A: pilot of the SLVC (CI A)

The SLVC pilot ran from 5 December 2023 to 6 June 2024. The SOP and referral criteria were shared with the cardiology team. Suitable patients already booked under the CLC model were reviewed and transferred to SLVC appointments.

During the pilot, Clinical Scientists drafted the patient and GP result letters, which were reviewed by a cardiologist for quality assurance. Across 45 SLVC cases, 93% were booked within one month of the guideline-recommended interval, with a mean earliness of 0.5 months. This exceeded the original baseline of 33% compliance and a mean earliness of 3.44 months under the CLC pathway (Fig. 3). Only three cases were booked substantially early; one involved a prosthetic valve with limited long-term durability data.

Result letter turnaround improved markedly, with SLVC letters averaging 1.4 working days to dispatch, compared to 8.3 days in the CLC model (Fig. 3, graph 3). Seven special cause outliers were excluded from the SPC chart analysis, including a run of delays due to staff sickness and overlapping annual leave. These vetting-related delays were anticipated to resolve, as vetting was only required for the pilot phase.

The SLVC met both primary QIP targets: improved surveillance interval adherence and reduced delay in result communication. No corresponding improvement was initially observed in the CLC group (under SPC rules [38]). This discrepancy likely reflected lower awareness of BSE guidance among CLC triaging staff and a reluctance to challenge established booking patterns. Or simply that this was a result of the more-complex caseload remaining on the CLC so many patients did not fit comfortably into a specific category. The pilot was continued with the aim of spreading better booking practice to the CLC.

#### PDSA B1-3: implementation and refinement of SOPs (CI B)

**Cycle B1** involved distributing the initial SOP to all relevant staff. As shown in Fig. 3, the proportion of CLC bookings within one month of the recommended interval improved from 33% to 85%, and mean earliness fell to 1.2 months. However, inconsistencies persisted, especially in moderate and mild valve disease cases.

**Cycle B2** introduced educational sessions to clarify BSE guidance on triaging moderate AS, particularly the use of Vmax criteria.

**Cycle B3** refined triage for patients with prosthetic valves of known durability. A reference document was shared with SLVC staff to guide appropriate follow-up intervals. As a result, erroneous bookings for these patients were significantly reduced, as demonstrated by a run of above-threshold values on the SPC G chart (Fig. 3, bottom).

#### JDI1: administrative refinement and electronic referral (CI C)

The administrative shift to short-horizon (6–8 week) booking and the implementation of electronic SLVC referrals reduced the likelihood of missed or duplicate requests. A minor issue emerged with referrals of ineligible patients, as referral letters were simultaneously sent to patients and the SLVC team. A rejection protocol was introduced to address this.

#### JDI2: duplicate booking identification (CI D)

The monthly duplicate booking search identified 15 patients (0.3%) with multiple TTE appointments between December 2023 and June 2024. All were addressed manually. Most errors originated from newly trained administrative staff, prompting the introduction of refresher training sessions. This process is now routinely performed to prevent waste and improve scheduling accuracy.

The PDSA cycles and results are summarised in Table 1; Fig. 3.

## Final Results

The four primary goals of this QIP, outlined in the driver diagram (Fig. 1), were assessed using OMs. These included: improved adherence to surveillance intervals (OM1), reduced result letter delays (OM2), fewer duplicate bookings (OM3), and lower per-patient service cost (OM4).

For OM1, the project achieved a combined post-intervention booking adherence rate of 88% (patients booked ≤ 1 month early), compared with 33% at baseline. The mean earliness was reduced to 0.99 months, just within the target of < 1 month. SLVC patients comprised 28% of all HVD surveillance slots during the pilot, lower than the 35% estimated as suitable for SLVC. Thus, projected aggregate benefits are likely conservative.

Regarding OM2, SLVC letters were dispatched within 5 working days in 93% of cases, with a mean turnaround time of 1.4 days. CLC remained slower, averaging over 8 days. Special cause analysis confirmed the SLVC pathway consistently met performance targets. The initial cardiologist-led letter review phase provided valuable training, with no clinical interpretation errors and progressive reduction in editorial changes over time.

As per other studies involving Clinical Scientist- or physiologist-led services [41], as long as there is a robust quality assurance process and governance regarding patients who require MDT input, there is no reason why these diagnostic tests and clinical assessments cannot be performed and reported by non-medical professionals. The SLVC model facilitated same-day communication of results to patients, which aligns with patient preference for rapid feedback [38] and contributes to satisfaction. Feedback forms distributed after SLVC appointments reported no material concerns.

For OM3, the new monthly duplicate booking search process identified and corrected 15 duplicate cases (0.3% of bookings). While baseline data were not quantifiable, the system now enables proactive monthly checks, eliminating this source of waste going forward. Errors were most commonly linked to new administrative staff, reinforcing the need for regular training.

OM4 compared per-patient service costs (Fig. 5). Based on current NHS tariffs, a CLC surveillance cycle (TTE + follow-up appointment) costs approximately £199. The SLVC model was estimated to deliver equivalent surveillance for 81% of that cost—a 19% saving. When combined with reductions in overprocessing, SLVC booking patients with lower earliness than if booked under CLC. Workload was also reduced through the SLVC (3 of 6 patients with mild HVD were discharged from SLVC, compared to none in CLC), the projected system-wide cost saving rose to 21%.

Patients should be discharged from routine follow up if they have mild aortic or mitral regurgitation but an anatomically normal valve structure and morphology and a normal aortic root, as per BSE guidelines [42]. The impact of all the above changes depends on the guideline surveillance booking intervals (so the type of patients in the casemix) as well as the earliness of appointments, so requires more-involved modelling.

Some inappropriate bookings persisted for prosthetic valve patients. Following SOP refinement, SPC G chart data (Fig. 3) indicated improvement, though further reinforcement may be required to fully embed best practices.

Overall, this QIP demonstrated measurable improvements in efficiency, cost-effectiveness, and consistency of HVD surveillance care. The results support the continued use and scaling of the SLVC pathway alongside refined triage protocols across CLC.

### Assessment of projected impact

**Fig. 8:**
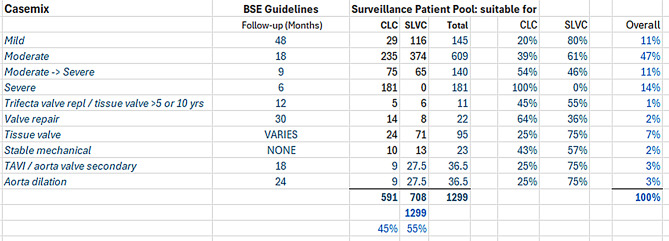
Surveillance pool model - Casemix

To assess the broader implications of the QIP, projected impacts were modelled using data from 36 baseline CLC patients, 95 improved CLC patients, and 45 SLVC patients. These numbers are sufficient for robust QIP analyses. Reducing the earliness of bookings (overprocessing in lean thinking terms) means each patient is being seen less frequently, which releases staff time to see other patients or do other work. These figures were applied to a snapshot of the HVD surveillance population at the Trust, totalling 1,299 patients. Ten diagnostic categories were defined, and the proportion of patients suitable for SLVC was estimated at 55% (Fig. 8).

Four scenarios were modelled:

- Scenario A: All patients managed under the original (baseline) CLC model.
- Scenario B: All patients managed under the improved post-QIP CLC model.
- Scenario C: All suitable patients managed under SLVC, remainder under improved CLC.
- Scenario D: As per Scenario C, but with perfect adherence to BSE surveillance intervals (zero earliness).

**Fig. 9:**
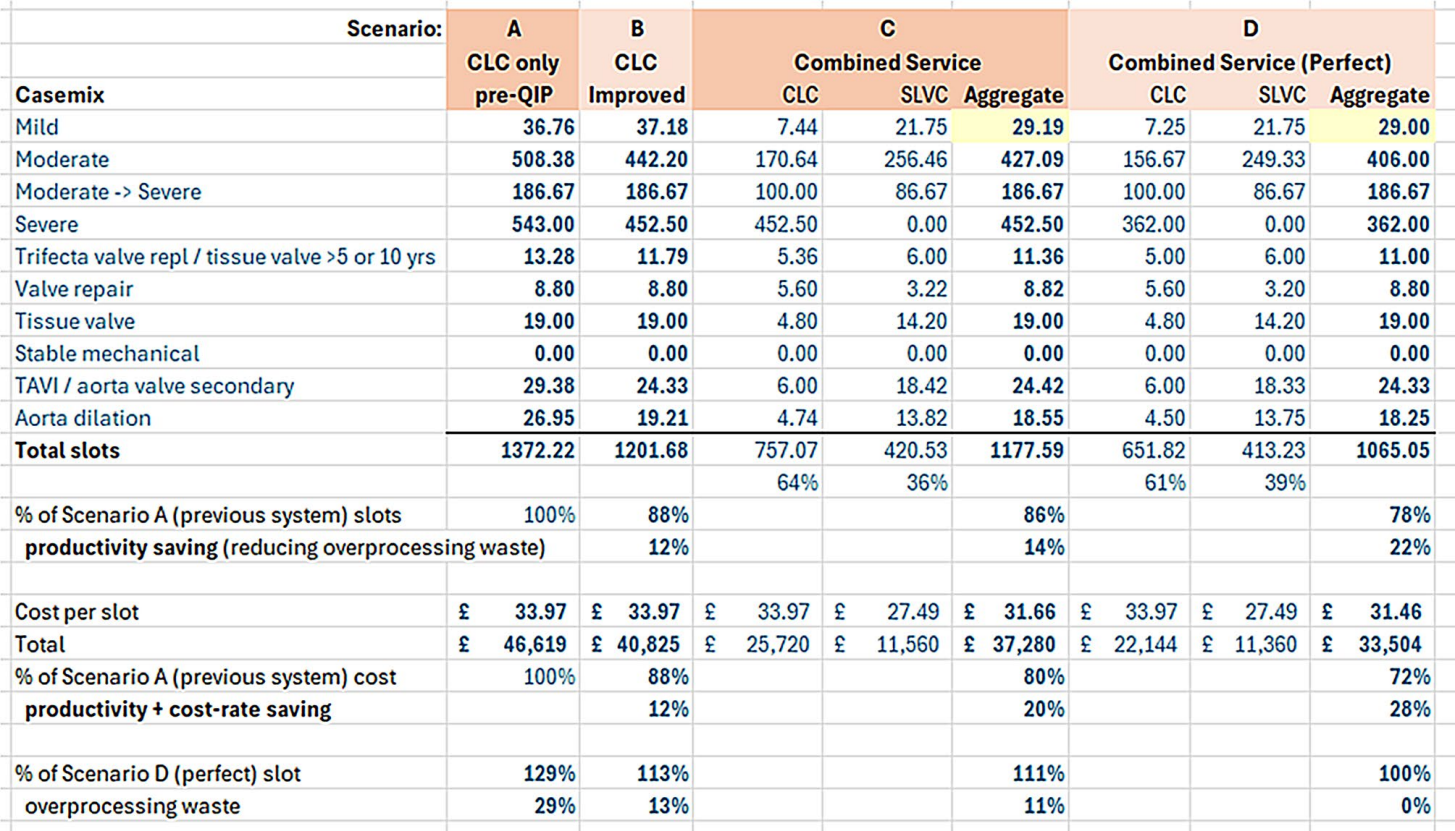
Surveillance pool model – Slots required per year

Results are summarised in Figs. 8 and 9.

In Scenario A, overprocessing, which is defined as the proportion of appointments booked earlier than guidelines recommend, was estimated at 29%. The QIP changes in Scenario B reduced this to 13%, and Scenario C reduced it further to 11%, due to better adherence in SLVC.

These reductions in overprocessing translate directly into increased system productivity. Compared with Scenario A, productivity improved by 12% in Scenario B and 14% in Scenario C. As SLVC handled only 28% of patients during the pilot, further productivity gains are expected if full SLVC deployment is achieved.

In Scenario C, residual overprocessing is due to severe cases still managed by CLC. Full implementation of triage SOPs and consistent application across staff groups may yield additional improvements.

The lower cost-per-patient of SLVC further amplifies efficiency gains. When cost differentials between SLVC and CLC are factored in, overall system cost reductions increase from 14% (productivity-driven) to 20%. This meets the original QIP goal of reducing HVD surveillance costs by more than 20%.

These findings underscore the scalability and sustainability of the intervention. Wider adoption could help address workforce pressures, reduce unnecessary appointments, and maintain or enhance care quality in an ageing patient population.

## Discussion

This QIP successfully demonstrated that care for patients with mild-to- less-than-severe HVD can be delivered more efficiently and consistently by implementing structured triage protocols and establishing a SLVC pathway. The project achieved measurable improvements in adherence to guideline-recommended surveillance intervals, reductions in delays communicating results, minimisation of duplicate bookings, and overall cost savings.

A key enabler of success was the collaborative approach involving Clinical Scientists, cardiologists, and administrative staff. While SOPs provided a framework for consistent triage decisions, the PDSA cycles revealed that education and reinforcement were essential for ensuring adherence. The improvement in CLC triaging, while not as strong as that in SLVC, was nonetheless substantial, demonstrating the benefit of targeted education sessions and shared protocols. Overprinting habits requires a period of practice and repetition [20, 43, 44]. 

Initial implementation of the SLVC was limited to a pilot phase, which required retrospective rebooking of patients from the CLC list. This introduced administrative complexity, particularly during periods of staff shortages. As such, some eligible patients remained in CLC to avoid the added burden of re-triaging and rebooking. A fully integrated electronic referral system, allowing precise categorisation and scheduling at the time of referral, would enhance sustainability and reduce administrative workload.

The success of the SLVC in improving efficiency and patient experience was bolstered by the structured vetting process, which helped Clinical Scientists refine their communication skills and clinical confidence. Over time, the need for cardiologist vetting diminished as confidence and consistency increased. The SLVC also enabled same-day communication of results, a known patient priority, which may contribute to improved satisfaction and engagement.

Since this QIP concluded, BSE guidance has evolved, particularly regarding surveillance intervals for moderate aortic stenosis. The shift to a unified 1–2-year follow-up for this group may streamline triage and further reduce variability. Similarly, updated guidance on prosthetic valve follow-up would support more consistent decision-making, particularly if valve-specific durability data were standardised in clinical tools. Chambers et al., describe when prosthetic heart valves should be followed up, determined by the timeframe since implant and the valve type [7]. However, whilst some examples of valves with proven longevity are given within that paper, it would be helpful to have a full list of time frames for each valve to refer to in clinic.

Future work could build on the current service model by incorporating stock-and-flow modelling of expected HVD case growth. With an ageing population and the progressive nature of HVD, planning for capacity needs and workforce development is essential. The spreadsheet model developed in this QIP offers a foundation for forecasting and business case development.

Overall, this QIP demonstrates the value of Clinical Scientist-led service models in cardiology. It highlights how a structured, MDT approach can optimise care pathways, improve resource use, and support workforce resilience.

## Conclusions

This QIP demonstrated that structured triage protocols and the implementation of a SLVC can enhance the efficiency, consistency, and cost-effectiveness of HVD surveillance services. By reducing premature surveillance bookings, improving adherence to BSE guidance, shortening result communication times, and minimising booking errors, the QIP achieved all four of its primary goals.

The SLVC model proved to be a safe, scalable, and low-cost alternative to CLC for appropriately selected patients, offering a more efficient use of clinical time and resources while maintaining a high standard of patient care. The process improvements documented here also contributed to workforce development by enabling Clinical Scientists to extend their clinical roles with appropriate training, oversight, and governance.

This work provides a replicable model for other NHS trusts aiming to meet increasing demand for echocardiographic surveillance in an ageing population. It also underscores the importance of using data-driven methods and MDT collaboration in designing sustainable service innovations. Broader adoption of these principles could support national workforce transformation goals while maintaining quality and safety in cardiology services.

It is hoped that this paper also serves to demonstrate the application and documentation of a rigorous QIP approach, with its supporting tools and techniques. These are part of the skillset that the NHS National School of Healthcare Science’s Higher *Specialist Scientist Training Programme* is building in the leadership of the clinical science workforce.

## Data Availability

The anonymised datasets used and/or analysed during the current study are available from the corresponding author on reasonable request.

## Abbreviations

AS: Aortic Stenosis
BSE: British Society of Echocardiography
CI: Change Idea
CLC: Cardiologist-Led Care
GP: General Practitioner
HCPC: Health and Care Professions Council
HVD: Heart Valve Disease
JDI: Just Do It!
MfI: Model for Improvement
MPH: Musgrove Park Hospital
NHS: National Health Service
OM: Outcome Metric
PDSA: Plan, Do, Study, Act
PM: Process Metric
QIP: Quality Improvement Project
SFT: Somerset Foundation Trust
SLVC: Clinical Scientist-Led Valve Clinic
SMART: Specific, Measurable, Achievable, Realistic, Timely
SOP: Standard Operating Procedure
SPC: Statistical Process Control
TTE: Transthoracic Echocardiography
Vmax: Peak Aortic Velocity
